# Beluga (*Delphinapterus leucas*) granulocytes and monocytes display variable responses to *in vitro* pressure exposures

**DOI:** 10.3389/fphys.2015.00128

**Published:** 2015-05-06

**Authors:** Laura A. Thompson, Tracy A. Romano

**Affiliations:** Research and Veterinary Services, Mystic Aquarium, A Division of Sea Research Foundation Inc.Mystic, CT, USA

**Keywords:** marine mammal, immune function, phagocytosis, diving, compression, decompression, whale, innate immunity

## Abstract

While it is widely known that marine mammals possess adaptations which allow them to make repetitive and extended dives to great depths without suffering ill effects seen in humans, the response of marine mammal immune cells to diving is unknown. Renewed interest in marine mammal dive physiology has arisen due to reports of decompression sickness-like symptoms and embolic damage in stranded and by-caught animals, and there is concern over whether anthropogenic activities can impact marine mammal health by disrupting adaptive dive responses and behavior. This work addresses the need for information concerning marine mammal immune function during diving by evaluating granulocyte and monocyte phagocytosis, and granulocyte activation in belugas (*n* = 4) in comparison with humans (*n* = 4), with and without *in vitro* pressure exposures. In addition, the potential for additional stressors to impact immune function was investigated by comparing the response of beluga cells to pressure between baseline and stressor conditions. Granulocyte and monocyte phagocytosis, as well as granulocyte activation, were compared between pressure exposed and non-exposed cells for each condition, between different pressure profiles and between conditions using mixed generalized linear models (α = 0.05). The effects of pressure varied between species as well by depth, compression/decompression rates, and length of exposures, and condition for belugas. Pressure induced changes in granulocyte and monocyte function in belugas could serve a protective function against dive-related pathologies and differences in the response between humans and belugas could reflect degrees of dive adaptation. The alteration of these responses during physiologically challenging conditions may increase the potential for dive-related in jury and disease in marine mammals.

## Introduction

The non-specific activity of the innate immune system is the first line of defense against pathogens, and plays an important role in wound healing. Innate immune responses aid in clearing infection and promotion of wound healing, yet abnormal cellular activity can lead to disease if (1) pathogens are not removed and destroyed, or (2) an inappropriate response occurs resulting in tissue damage (Smith, 1994). In humans, alterations in immune function resulting from the challenges associated with diving can lead to increased incidents of dive related injury and disease (Brenner et al., 1999). For example, development of decompression sickness (DCS) is associated with immune activity and inflammatory damage in particular, resulting directly or indirectly from the formation of gas bubbles in blood and tissues (Ward et al., 1987; Nyquist et al., 2004; Barack and Katz, 2005). Symptoms are variable, with some resembling anaphylaxis and complement activation (Montcalm-Smith et al., 2007) suggesting involvement of the immune system. Activation of the alternate complement pathway, involving binding of CR3, has been reported to be indicative of sensitivity to DCS development (Ward et al., 1987). In addition, aggregation of leukocytes in tissue such as the lung and liver, accompanied by cell activation are a marker of DCS in pigs (Nyquist et al., 2004). Less dramatic symptoms of mild DCS include pruritus or itching of the skin related to an inflammatory response.

It has long been thought that marine mammals are not subject to decompression related injuries and the physiological and behavioral adaptations that allow marine mammals to make extended and repetitive dives to great depths without suffering injury have been the subject of great interest. For example, breath hold diving and physiological responses, such as lung collapse, have been implicated in protecting marine mammals from developing decompression sickness, unlike human scuba divers who need to carry an air supply with them. However, DCS, or DCS like emboli, and symptoms can occur during breath hold diving if the characteristics of the dive do not allow appropriate gas washout (Paulev, 1967; Ferrigno and Lundgren, 2003; Wong, 2006), and tissue nitrogen loads have been estimated to reach 200–300% in bottlenose dolphins (*Tursiops truncatus*) and beaked whales (Houser et al., 2001; Jepson et al., 2003). It is possible that marine mammals normally face conditions that result in DCS in people, but have developed protective adaptations to avoid it. One mechanism by which marine mammals can resist damage from bubbles may be a less reactive immune response (Ward et al., 1987; Fahlman et al., 2006).

In recent years there have been several reports of gas bubbles and associated injury in several marine mammal species including beaked whales which stranded in close proximity to naval sonar excercises (Jepson et al., 2003; Fernandez et al., 2005) stranded dolphins (Dennison et al., 2012) and several species of marine mammals which became entangled in fishing gear at depth and were subsequently brought to the surface rapidly (Moore et al., 2009). In each of these cases, rapid decompression has been a suggested etiology of bubble formation, but there is concern that human activities, such as sonar use, play a role, potentially by interrupting natural adaptation to environmental challenges, either through altering behavior or acting as a stressor. For example, deep diving beaked whales often perform short, shallow bounce dives following prolonged exposures to great depth, which have been suggested to aid in safe off gassing of high nitrogen loads (Tyack et al., 2006). Avoidance behaviors, or a startle response, triggered by human activity may result in altered duration of dives or swim speeds, which may then increase the potential for bubble formation. Moreover, release of catecholamines or glucocorticoids occurs during a stress response. Romano et al. (2004) reported increased epinephrine, norepinephrine and dopamine following exposure to loud sound in a bottlenose dolphin and beluga whale (*Delphinapterus leucas*). These hormones can modulate immune function through adrenergic and adrenal steroid receptors expressed on cells (Madden et al., 1995; McEwen et al., 1997; Padgett and Glaser, 2003) and the effect on marine mammal health may be subtle, but cumulative over repeated exposures.

Dive studies, however, are limited to relatively few species, and cellular adaptations have received little attention. Changes in pressure have been noted to affect multiple aspects of cell function (reviewed in Macdonald, 1982; Heinemann et al., 1987; Somero, 1992; Bartlett, 2002; Daniels and Grossman, 2003; Pradillon and Gaill, 2007). Many of these effects are likely mediated through changes in membrane characteristics (Macdonald, 1982; Kato and Hayashi, 1999) and reducing the volume in which molecules interact with each other (Bartlett, 2002). For example, ordering of membrane structures occurs under increased pressure resulting in reduced membrane fluidity as well as membrane volume (Macdonald, 1982; Somero, 1992). In addition, membrane related processes and membrane associated proteins are secondarily affected (Macdonald, 1982; Somero, 1992). Furthermore, gene expression and protein synthesis have been shown to be altered by high pressure (Bartlett et al., 1995; Pradillon and Gaill, 2007). Components of the cytoskeleton can be reorganized and actin has been reported to undergo pressure induced de-polymerization (Haskin and Cameron, 1993).

Whereas differences in the response of platelets and red blood cells to increased pressure have been noted between marine mammals and humans or other terrestrial species (Field, 2000; Castellini et al., 2001; Williams et al., 2001), there have been no published reports concerning potential impact of diving on immune function in marine mammals.

Field (2000) investigated the effects of pressure and chilling on platelet activation in humans and the northern elephant seal (*Mirounga angustirostris*). Pressures of 2800 psi (corresponding to a depth of ~2000 m) did not induce activation in the elephant seal platelets, while human platelets displayed shape changes indicative of activation. These differences indicate that elephant seal platelets are better suited for handling exposure to pressure, and the author describes the membrane cholesterol content as a potential mechanism.

Red blood cell metabolism of marine mammals during simulated exposures to 2000 psi has been compared between shallow and deep diving species, as well as with non-diving terrestrial mammals (Castellini et al., 2001). Glycolytic activity, calculated as a measure of cell metabolism in this study, tended to increase in marine mammal cells, while the response of terrestrial mammal cells was much more variable. Among the marine mammals, the largest changes occurred in shallow diving phocid species such as harbor seals (*Phoca vitulina*), suggesting a greater sensitivity to the effects of diving than in species which regularly make deep dives. Further investigations have shown erythrocytes from deeper diving species, such as elephant seals, have different fatty acid chain and cholesterol content in their membranes as compared to terrestrial mammals such as cows, horses and dogs, while shallow diving species had membrane compositions more similar to terrestrial mammals (Williams et al., 2001).

The purpose of this study was to investigate the functional response of granulocytes and monocytes to simulated diving in belugas (*Delphinapterus leucas*) during baseline and stressor conditions. Belugas have been reported to possess a repertoire of dive patterns (Martin et al., 1998) and are capable of diving to depths of 900 m and more (Heide-Jorgensen et al., 1998; Suydam et al., 2001). Granulocyte and monocyte phagocytosis, as well as granulocyte activation were measured with and without exposure to increased pressure *in vitro*. These are important cells of the innate immune response, and inefficient or inappropriate responses in these cells can lead to disease and self-injury. The process of phagocytosis involves aggregation of membrane receptors (Aderem and Underhill, 1999; Greenberg, 1999), development of pseudopodia, as well as membrane invaginations and membrane fusion (Murphy et al., 2008), all of which may be altered by pressure effects on cell membrane processes (Macdonald, 1982; Heinemann et al., 1987; Somero, 1992; Haskin and Cameron, 1993; Kato and Hayashi, 1999).

Adaptation of beluga cells was assessed by comparing baseline results with those measured in human blood samples, with the hypothesis that human cells would display altered function in response to changes in pressure but that beluga cells would not. The effects of additional “stressors” on the function on beluga cells was evaluated using samples drawn following a 30 min out of water examination (OWE) or during a period of physiological challenge (i.e., inflammation). The results of this study are intended as a first look at immune function in relation to diving in a marine mammal species, and provide information concerning the potential impacts of multiple challenges on marine mammal health.

## Methods

### Animal subjects and samples

Blood samples were obtained from four belugas (two females ~30 years old and two males ~25 and 9 years old) resident at the Mystic Aquarium, Mystic, CT. For baseline samples, animals were trained to present the ventral aspect of the flukes for blood collection. Stressor samples were obtained from 3 animals following a 30 min out of water examination (OWE) or from 2 animals presenting with clinical signs of inflammation. During the OWE, animals were stretchered and placed upon the exhibit beach for examination. Blood samples were drawn from the dorsal aspect of the flukes following the 30 min examination and prior to return of the animals back into the water. For the 2 whales presenting with inflammation, blood samples were obtained during follow-up health checks and were opportunistically used to further evaluate the potential role of a physiological challenge in altering immune function and the immune response to changes in pressure. Blood was drawn in 10 ml sodium heparin vacutainer™ tubes and placed on ice (Mystic Aquarium IACUC protocol No. 11001; UConn IACUC reciprocation No. R12-002). Human blood samples were purchased from Biological Specialty Corporation (www.biospecialty.com) and shipped on ice packs within 24 h. Both phagocytosis and granulocyte activation assays were performed on fresh whole blood samples. Remaining sample (10–20 ml) was centrifuged at 2000 × g and 10°C for 10 min in order to isolate plasma and the white blood cell buffy coat for archiving. Plasma was removed and stored in 1.5 ml Sarstedt™ tubes, immediately placed on ice and transferred to −80°C for storage. Buffy coats were mixed with an equal volume of freezing media (90% Fetal Bovine serum and 10% DMSO), slowly frozen at −80°C over 24 h and transferred to liquid nitrogen for future assays.

### Simulated pressure excursions

Blood samples as well as the internal temperature of the pressure chamber were brought to 37°C. Four ml of blood were added to the pressure chamber through a top loading port, and over-laid with a thin layer of mineral oil. The loading port was closed off and mineral oil pumped into the chamber by hand using a hydraulic pump, rated to 40,000 psi, in order to pressurize the sample. Mineral oil has been used in previous studies to pressurize biological samples (e.g., Curl and Jansen, 1950; Somero et al., 1977; Field, 2000) and is not biologically reactive and so should not interfere with measurements of immune function targeted by this study. Preliminary experiments were run exposing 4 ml of blood to mineral oil (without pressure) and comparing immune function measurements with blood not exposed to mineral oil with no significant changes.

A pressure gauge was used to monitor the rate of compression and decompression, as well as to maintain the sample at the desired pressure. At the conclusion of a pressure excursion, pressure was released by hand by loosening valve connections between the pressure chamber and oil pump. Blood samples were then removed using a sterile transfer pipette and aliquoted into FACS™ (BD Biosciences, San Jose, CA) tubes as per assay descriptions below.

Targeted dive profiles are summarized in Table 1. Three durations were used; a single 30 min dive, a single 5 min dive, and two 5 min dives with a 1 min “rest” period. These durations were chosen to represent both a prolonged dive (30 min) and more common shorter dives (5 min). While the majority of dives performed by belugas last around 10 min or less, they have been reported to stay submerged for longer than 20 min (Shaffer et al., 1997; Martin and Smith, 1999). Target simulated depths were 1360 m (2000 psi) and 680 m (1000 psi). These pressures represent deep dives which would push the physiological limits of an animal. Compression and decompression occurred gradually (G) over a period of either 2 min or rapidly (R) over 15 s. While these rates are faster than would occur in a diving beluga, they were chosen due to restrictions of the pressure system. These two rates could be reproduced with minimal variation, as compared with compression/decompression over longer periods. Due to sampling restrictions, OWE samples were only exposed to 2000G and inflammation samples were only exposed to 2000G and 1000G.

**Table 1:** **Dive profiles targeted for pressure exposures**.

**Pressure**	**Simulated depth**	**Compression/Decompression**	**Duration (min)**
2000 psi	1360 m	2 min (2000G)	30 5 2 × 5
2000 psi	1360 m	15 s (2000R)	30 5 2 × 5
1000 psi	680 m	2 min (1000G)	30 5 2 × 5
1000 psi	680 m	15 s (1000R)	30 5 2 × 5

*Two depths were chosen with exposures lasting for 3 different durations. Compression and decompression were varied at 2 min (G) or 15 s (R)*.

### Phagocytosis

Phagocytic activity was measured via flow cytometry using propidium iodide labeled Staphylococcus aureus (PI staph) based on the protocol from Spoon and Romano (2012). Slight modifications to this protocol were made to include simulated dive exposures.

For both belugas and humans, white blood cell counts were obtained using Trypan blue exclusion, and average cell counts were used to calculate the required volume of stock PI Staph (4.8 × 10^9^ ml^−1^) to obtain a bacteria: cell ratio of 25:1. One hundred μl of whole blood were added to FACS™ tubes for controls, with 4 ml of blood set aside to introduce to the pressure chamber as described above. Ten and 400 μl of PI Staph solution were added to controls and 4 ml of blood, respectively. At the time the bacteria were added, the 4 ml of blood were introduced to the pressure chamber and all samples were then allowed to incubate for the duration of the simulated dive excursion. Control tubes were run simultaneously with pressure experiments but without pressure exposure, and included cells only and PI Staph fluorescence controls. At the conclusion of the dive excursion, 100 μl of pressure exposed blood were aliquoted into FACS™ tubes. In order to stop cell activity, 10 μl of 10 mM N-ethylmaleimide were added immediately following the conclusion of each simulated dive excursion (dive period) or after an additional 20 min (recovery). Tubes were then incubated on ice until lysis (up to 1 h). Two ml of lysis buffer (0.01 M Tris; 0.001 M EDTA; 0.17 M NH_4_Cl solution; pH 7.4) were added to each tube and incubated for ~15 min to lyse red blood cells. Remaining white cells were then washed twice with 1 ml of 1x PBS and centrifuged at 220 × g for 3 min, before fixing in 250 μl of 1% paraformaldehyde. Tubes were stored at 4°C, in the dark, until analyzed by flow cytometry 24 h later.

### CD11b phenotyping

A mouse anti-canine CD11b IgG1 was used to measure granulocyte activation. This antibody has previously been used in cetaceans (De Guise et al., 2004). In addition, our lab demonstrated cross reactivity by performing both a western blot and PMA stimulation tests with beluga cells. Due to limitations of the equipment being used, evaluation of CD11b expression was run on the same samples as the phagocytosis assay. As such, pressurized experimental cells were exposed to PI Staph during the pressure exposures and prior to the CD11b assay. In order to keep consistency between controls and experimental samples, non-pressure exposure cells were also incubated with PI Staph for an equivalent amount of time, prior to the CD11b assay.

Following simulated dive excursions, 100 μl of either non-exposed or pressure exposed blood were aliquoted into FACS™ tubes, and 50 μl of Mouse Anti-Canine CD11b IgG1 (AbD Serotec, Raleigh, NC) diluted 1:5 in Hank's Balanced Salt Solution (HBSS) was added. Phorbol myristate acetate (PMA) stimulation was used to verify cross reactivity and behavior of this antibody and its target. Blank controls received 50 μl of HBSS, and negative controls received 50 μl of Mouse IgG (Sigma, St Louis, MO). Cells were incubated for a further 30 min at 37°C, washed twice with 4°C HBSS and centrifuged at 220 × g for 5 min. Cells were then incubated in the dark at 4°C with a 1:10,000 dilution of fluorescein isothiocyanate (FITC) labeled goat anti-mouse IgG (Beckman Coulter, Miami, FL) for 30 min, washed twice with cold HBSS and placed on ice. Red blood cells were subsequently lysed in the same manner as reported above for the phagocytosis assay. Tubes were fixed in 250 μl of 1% paraformaldehyde and stored in the dark at 4°C until analyzed by flow cytometry within 24 h.

### Flow cytometry

Samples were read using a LSR flow cytometer (BD Biosciences, San Jose, CA) and cell quest software for analysis. Forward and side scatter plots were obtained from tubes containing cells only and used to gate the cell populations of interest. The propidium iodide (phagocytosis) and FITC (CD11b) signals were read at emission wavelengths of 617 and 518 nm respectively and were detected in FL2 and FL1 channels respectively.

For phagocytosis data, both the granulocyte population, composed mostly of neutrophils, and monocyte population were gated for data collection. One hundred thousand total events were collected (Spoon and Romano, 2012). Results reported here are the mean intensity of the fluorescence (MFI) expressed by the population, reflecting how many bacteria have been ingested per cell. Only the granulocyte population was targeted for data collection for expression of CD11b, and results are reported as the percentage of the target population which express the FITC fluorescence (i.e., are expressing CD11b). Ten thousand granulocytes were collected.

### Hormone analysis

Plasma concentrations of cortisol as well as the catecholamines epinephrine and norepinephrine were measured in blood samples to demonstrate a physiological change from baseline for stressor conditions. One ml of plasma was shipped to the AHDC Endocrinology Lab at Cornell University (Ithaca, NY) for determination of cortisol concentration. Catecholamines were measured in-house using a Waters (Milford, MA) High Performance Liquid Chromatography systems (1515 isocratic pump, 717 auto sampler) with electrochemical detection (2465 electrochemical detector). The methodology for this has been detailed in “Plasma Catecholamines by HPLC” (Instruction Manual, June 2001, BIO-RAD, Hercules, CA). Two ml of sodium plasma were thawed and added to 50 mg of acid washed alumina (BioRad Cat. 195–6055). Two ml of 0.1 M H_3_PO_4_, Calibrator (BioRad) and high and low controls (BioRad) were also run. Two hundred ul of internal standard and 2 ml of 1 M Tris buffer (pH 6.8) were added to each tube, mixed and placed on a rotor at 75% for 19 min. Samples were then centrifuged and washed twice with 1 ml water before the addition of 200 ul of 0.1 M H_3_PO_4_. Samples were centrifuged at 2100 × g and 5°C for 5 min after which the supernatant was transferred to autosampler vials and loaded into the HPLC system for analysis.

### Statistics

Measures of immune function following pressure exposures were normalized to control values by division and comparisons were run to determine: (1) if pressure exposed cells function differently from non-exposed cells (2) if pressure exposed cells function differently in response to different dive characteristics such as depth or duration, (3) if beluga cells respond differently to pressure exposures than human cells, and (4) if beluga cells respond differently to pressure during baseline and stressor conditions. Mixed effects generalized linear models, with repeated measures, were run for the dive and recovery periods for each pressure exposure. Individuals were entered as a random factor in the models, with species (beluga or human) and treatment (control or pressure exposed) entered as fixed factors. For each exposure duration comparisons were also made between each dive profile. For all comparisons α = 0.05. However, due to small sample sizes trends are also reported where α = 0.1. No statistics could be run on data collected from inflammation samples due to the sample size (*n* = 2). Hormones were compared between OWE and baseline conditions using repeated measures paired *T*-Tests (α = 0.05).

## Results

### Effects of pressure on baseline beluga samples

#### Effects of 2000 psi with 2 min compression/decompression (2000G) on phagocytosis and CD11b

Baseline beluga samples displayed decreases in phagocytosis for the dive periods of the 30 min (Granulocytes, *p* < 0.001) and single 5 min exposures (Granulocytes, *p* < 0.001; Monocytes, *p* = 0.03). Following the recovery period of the 30 min exposure the amount of activity occurring per cell displayed a trend of decreased function for granulocytes while no significant changes in monocyte function were detected for the recovery periods of any pressure exposures. Results are summarized in Table 2.

**Table 2:**
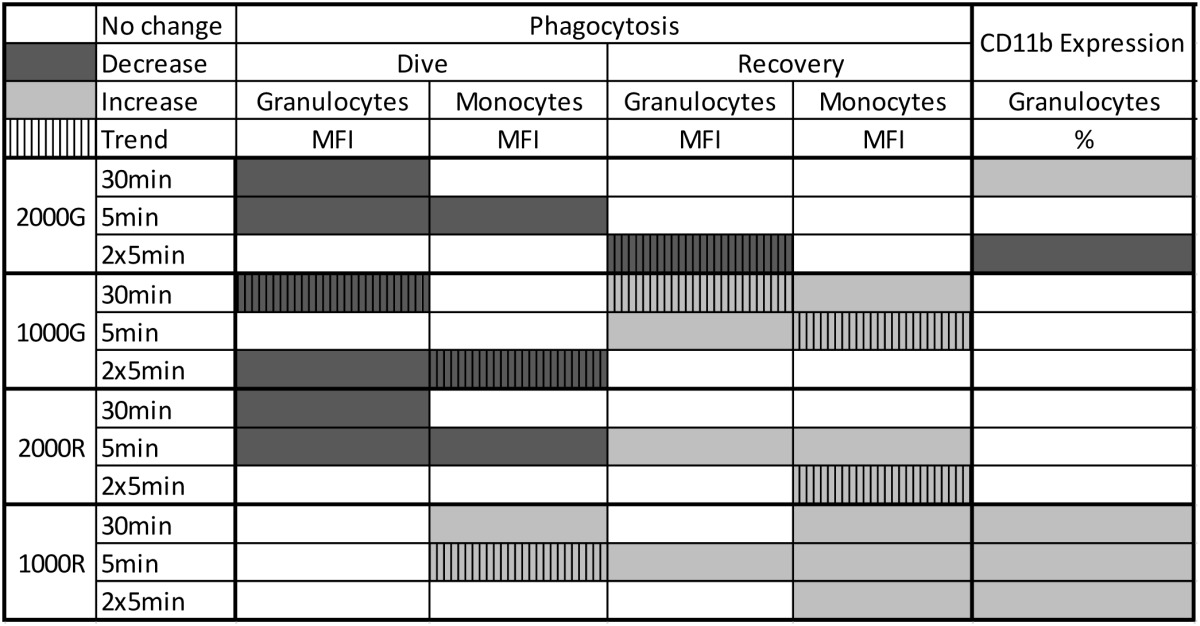
**Summary of results for Phagocytosis and CD11b Expression following simulated pressure exposures for baseline beluga samples**.

*Significant pressure induced decreases are indicated by dark grey boxes, while significant pressure induced increases are indicated by light grey boxes (p < 0.05). Vertical striping indicates trends in data (p < 0.1). White boxes indicate no significant changes or trends*.

Even though phagocytosis decreased, belugas displayed an increase in the % of cells expressing CD11b following the 30 min exposure (Table 2; *p* = 0.048). Significant decreases in the % of cells expressing CD11b were detected following the repeated 5 min exposures (Table 2; % Positive, *p* < 0.001).

#### Effects of 1000 psi with 2 min compression/decompression (1000G) on phagocytosis and CD11b

Beluga samples displayed decreased phagocytosis following the dive periods of the 30 min (Granulocytes, *p* = 0.074) and repeated 5 min (Granulocytes, *p* = 0.007; Monocytes, *p* = 0.093) exposures (Table 2). Increased phagocytosis however, was measured following the recovery periods of the 30 min (Granulocytes, *p* = 0.055; Monocytes, *p* = 0.023) and single 5 min exposures (Granulocytes, *p* = 0.019; Monocytes, *p* = 0.069). No significant changes in CD11b expression were detected for baseline beluga samples. Results are summarized in Table 2.

#### Effects of 2000 psi with 15 s compression/decompression (2000R) on phagocytosis and CD11b

Pressure induced decreases in phagocytosis were detected for the dive periods of both the 30 min (Granulocytes, *p* = 0.023) and single 5 min (Granulocytes, *p* < 0.001; Monocytes, *p* = 0.002) exposures (Table 2). In contrast, increased phagocytic activity was measured following the recovery periods of the 5 min exposure (Granulocytes, *p* < 0.001; Monocytes, *p* < 0.001) and repeated 5 min exposures (Monocytes, *p* = 0.056). No significant changes in CD11b expression were detected (Table 2).

#### Effects of 1000 psi with 15 s compression/decompression (1000R) on phagocytosis and CD11b

No changes in phagocytic activity were detected for beluga granulocytes for any dive period, though monocytes displayed increased function for the dive periods of both the 30 min (*p* < 0.001), and single 5 min (*p* = 0.087) exposures. Increased phagocytic activity was also detected for granulocytes following the recovery period of the 5 min (MFI, *p* = 0.011) exposures. Beluga monocytes also displayed increased activity following the recovery periods of the 30 min (MFI, *p* < 0.001) single 5 min (MFI, *p* = 0.003) and repeated 5 min (MFI, *p* = 0.012) exposures. Results are summarized in Table 2.

Significant increases in the % of granulocytes expressing CD11b were detected in belugas following all exposures to 1000R and results are summarized in Table 2 (30 min, *p* = 0.032; 5 min, *p* = 0.010; 2 × 5 min, *p* = 0.002).

### Comparative effects of pressure between dive exposures

#### Granulocyte phagocytosis

**Figure 1:**
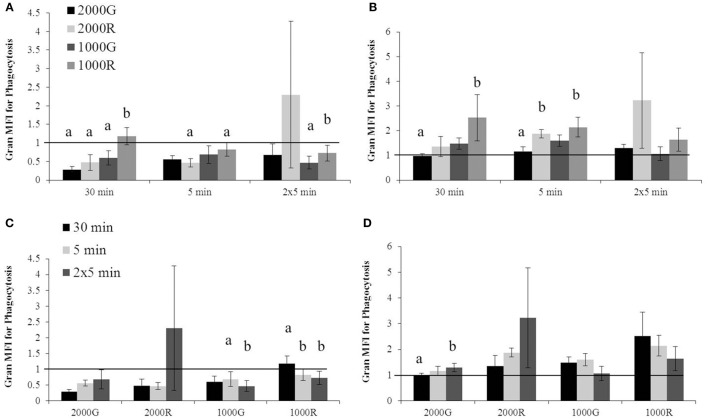
**Changes in granulocyte phagocytosis measured in baseline beluga samples (*n* = 4) for all dive exposures**. Results are compared between target pressures for both dive **(A)** and recovery **(B)** periods, as well as between durations for both dive **(C)** and recovery **(D)** periods. Data have been normalized to controls (represented by the solid line at 1) and presented as the mean change in function ± SE. Values greater than 1 indicate increased function), and values less than 1 indicate decreased function following pressure exposures (e.g., value of 2 indicates a 100% increase in function; a value of 0.5 indicates a 50% decrease in function). Significant differences between exposures (*p* < 0.05) are indicated by letters.

For dive periods, belugas displayed significantly smaller change in phagocytosis following exposure to 1000 R as compared with all other exposures for the 30 min duration (Figure 1A; 1000R vs. 1000G, *p* = 0.001; 1000R vs. 2000G, *p* = 0.001; 1000R vs. 2000R, *p* = 0.022). A significantly smaller response was also detected for the repeated 5 min exposure to 1000R as compared with 1000G (Figure 1A; *p* < 0.001), and for the single 5 min exposure to 1000R as compared with 2000R (Figure 1A; *p* = 0.093).

Following recovery periods (Figure 1B), exposures to 1000R resulted in a larger change in phagocytosis as compared with 2000G for the 30 and 5 min durations (30 min, *p* = 0.006; 5 min, *p* = 0.048). In addition, significantly greater changes in phagocytosis were detected following exposure to 2000R as compared with 2000G following the single 5 min durations (*p* = 0.028).

A significantly larger change in phagocytosis was detected following the repeated 5 min exposure to 1000G as compared with the single 5 min exposure (Figure 1C; *p* = 0.021). Both the single and repeated 5 min exposures to 1000R resulted in significantly larger changes in phagocytosis than the 30 min exposure (Figure 1C; 30 min vs. 5 min, *p* = 0.024; 30 min vs. 2 × 5 min, *p* = 0.006). For recovery periods (Figure 1D), the repeated 5 min duration dives resulted in larger responses in phagocytosis following exposures to 2000G as compared with 30 min duration exposures (*p* = 0.001).

#### Monocyte phagocytosis

**Figure 2:**
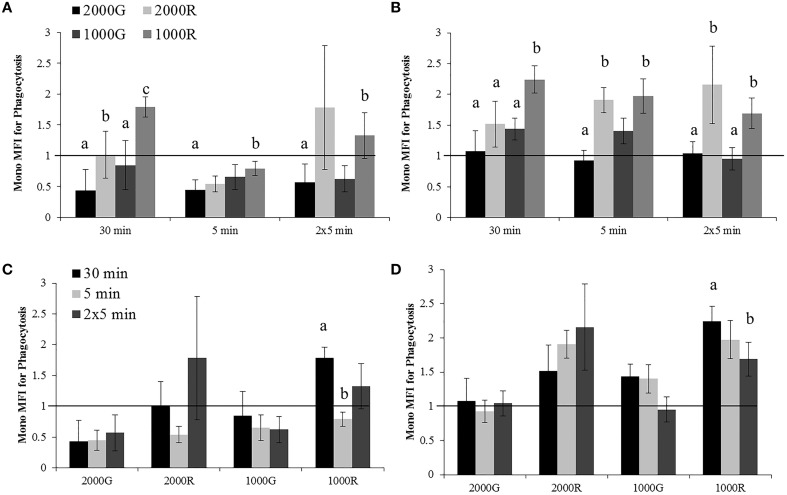
**Changes in monocyte phagocytosis measured in baseline beluga samples (*n* = 4) for all dive exposures**. Results are compared between target pressures for both dive **(A)** and recovery **(B)** periods, as well as between durations for both dive **(C)** and recovery **(D)** periods. Data have been normalized to controls (represented by the solid line at 1) and presented as the mean change in function ± SE. Values greater than 1 indicate increased function, and values less than 1 indicate decreased function following pressure exposures. Significant differences between exposures (*p* < 0.05) are indicated by letters.

The dive period of the 30 min exposures to 1000R resulted in significantly larger changes in phagocytosis as compared to all other exposures of the same duration (Figure 2A; 1000R vs. 1000G, *p* = 0.006; 1000R vs. 2000G, *p* < 0.001; 1000R vs. 2000R, *p* = 0.028). In this case, the response of cells to 1000R was an increase in function, whereas a decrease was observed in most other cases. Exposures to 1000R resulted in significantly smaller changes in monocyte phagocytosis as compared with 2000G for both the single 5 min (*p* = 0.001) and repeated 5 min durations (*p* = 0.082). Pressure induced changes in phagocytosis were also significantly larger for the 30 min exposures to 2000G as compared with 2000R (Figure 2A; *p* < 0.001).

Pressure induced changes in monocyte phagocytosis were significantly larger following the recovery period for exposures to 1000R as compared with all other exposures for the 30 min exposures (1000R vs. 1000G, *p* = 0.010; 1000R vs. 2000G, *p* < 0.001; 1000R vs. 2000R, *p* = 0.031), and from 2000G (*p* = 0.005) and 1000G (*p* = 0.011) for the repeated 5 min durations (Figure 2B). Additionally, monocytes displayed significantly different changes for exposures to 2000G as compared with dives of rapid compression and decompression for the single 5 min exposures (2000G vs. 1000R, *p* < 0.001; 2000G vs. 2000R, *p* < 0.001).

Effects of exposure duration on the response of monocyte phagocytic activity were only detected for 1000R exposures (Figures 2C,D). For the dive period, the response following the 30 min exposures was significantly larger than the single 5 min exposures (*p* < 0.001), while following the recovery period, the 30 min exposures resulted in larger changes than the repeated 5 min exposures (*p* = 0.05).

#### Granulocyte CD11b expression

**Figure 3:**
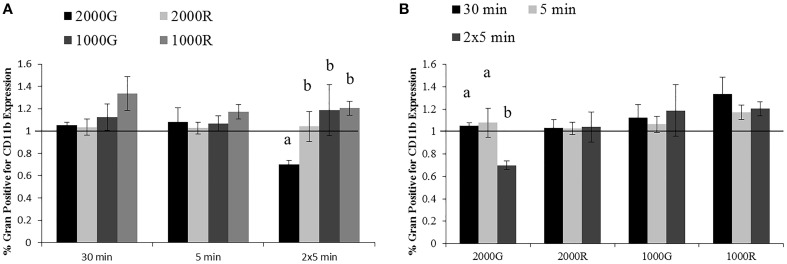
**Changes in CD11b expression measured in baseline beluga samples (*n* = 4) for all dive exposures**. Results are compared between target pressures **(A)** and exposure durations **(B)**. Data have been normalized to controls (represented by the solid line at 1) and presented as the mean in function ± SE. Values greater than 1 indicate increased function, and values less than 1 indicate decreased function following pressure exposures (e.g., a value of 2 indicates a 100% increase in expression, while a value of 0.5 indicates a 50% decrease in expression). Significant differences between exposures (*p* < 0.05) are indicated by letters.

A significantly larger change in granulocyte activation was detected following exposure to 2000G as compared with all other exposures for the dive period of repeated 5 min durations (Figure 3A; 2000G vs. 1000G, *p* = 0.050; 2000G vs. 2000R, *p* = 0.019; 2000G vs. 1000R, *p* < 0.001). Pressure induced changes in the % of positive cells were also larger following the 5 min exposure to 1000R as compared with 2000R (*p* = 0.061).

Additionally, the % granulocytes positive for CD11b expression showed a significantly larger decrease following repeated 5 min exposures to 2000G as compared with the changes measured for the 30 min and single 5 min exposures (Figure 3B; 2 × 5 min vs. 30 min, *p* < 0.001; 2 × 5 min vs. 5 min, *p* = 0.012).

### The effects of pressure on beluga OWE and inflammation samples

#### Hormone concentrations

Catecholamine and cortisol concentrations for baseline, OWE and inflammation conditions are summarized in Table 3. A significant increase was detected only for OWE cortisol as compared with baseline cortisol (*T* = −5.794; *p* = 0.029) though higher epinephrine values were also observed. A slight increase in norepinephrine was also observed for OWE and inflammation conditions though this was not significant.

**Table 3:** **Plasma catecholamines and cortisol (±SE) for belugas during baseline, OWE and inflammation conditions**.

		**Epinephrine pg ml-1**	**Norepinephrine pg ml-1**	**Cortisol μg dl-1**
Belugas	Baseline *n* = 4	<30	662.96 ± 110.5	1.57 ± 0.2
	OWE *n* = 3	78.02 ± 43.4	757.06 ± 81.4	7.97 ± 1.2^*^
	Inflammation *n* = 2	<30	780.38	1.97

Values which are significantly different from baseline (p < 0.05) are indicated with an asterisk (

* *)*.

#### Effects of 2000 psi with 2 min compression/decompression (2000G) on phagocytosis and CD11b expression

Significant decreases in phagocytosis were detected in OWE samples following the dive period of all exposures to 2000G for either granulocytes (30 min, *p* < 0.001; 5 min, *p* < 0.001) or monocytes (*p* < 0.001 for all cases). Following the recovery period of the 30 min exposure, however increased phagocytosis was detected for both granulocytes (*p* = 0.011) and monocytes (*p* = 0.051). While significance was not detected large increases were also observed following the 5 min and repeated 5 min exposures.

For inflammation samples, all pressure exposures to 2000G resulted in decreased granulocyte MFI during the dive periods, Decreased function was also observed for monocytes following the dive periods of the single and repeated 5 min exposures. Following the recovery period of the single 5 min exposure increased phagocytosis was observed for monocytes.

No significant effects of pressure were detected in OWE samples for CD11b expression for any pressure exposure. However, patterns suggestive of an increase in expression were observed for the repeated 5 min exposures (*p* = 0.096). Similarly no apparent changes in % of granulocytes positive for CD11b expression were observed for inflammation samples following exposures to 2000G.

#### Effects of 1000 psi with 2 min compression/decompression (1000G) on phagocytosis and CD11b expression

For exposures to 1000G, inflammation samples displayed decreases in granulocyte phagocytosis for the dive periods of all duration exposures. Monocytes also displayed decreased measures of phagocytosis for the dive periods of all exposures, though these values appeared to return to control levels for all recovery periods.

No apparent changes in for the % of positive granulocytes for CD11b expression were observed for inflammation samples following exposures to 1000G.

### Comparative effects of pressure exposures on beluga baseline, OWE and inflammation samples, and humans

#### Effects of 2000 psi with 2 min compression/decompression (2000G) on phagocytosis

**Figure 4:**
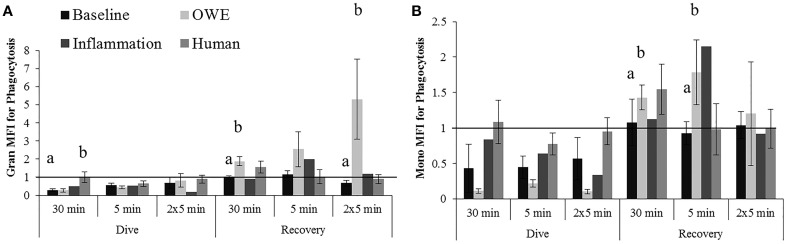
**Changes in Granulocyte (A) and Monocyte (B) phagocytosis following exposures to 2000G, measured in belugas under baseline (*n* = 4), OWE (*n* = 3) and inflammation (*n* = 2) conditions, and humans (*n* = 4)**. Data have been normalized to controls (represented by the solid line at 1) and presented as the mean change in function ± SE. Values greater than 1 indicate increased function, and values less than 1 indicate decreased function following pressure exposures. Significant differences between belugas and humans, or between conditions in belugas are indicated with letters (*p* < 0.05).

Humans displayed a significantly smaller change in granulocyte phagocytosis than belugas (baseline) for the dive period of the 30 min exposure (Figure 4A; *p* = 0.036). The response of beluga granulocytes from OWE conditions to pressure also differed from the response of human cells for this exposure (*p* = 0.025). Inflammation samples appeared to show a greater decrease in granulocyte phagocytosis than both baseline beluga and human samples for the dive period of the repeated 5 min exposures (Figure 4A).

For the recovery periods, OWE conditions showed a significantly larger increase in granulocyte phagocytosis than baseline beluga samples for the 30 min (*p* = 0.001) and repeated 5 min (*p* = 0.09) exposures. Inflammation samples displayed an apparently larger increase in granulocyte phagocytosis for the single 5 min exposure than both baseline and human exposures (Figure 4A).

The change in monocyte phagocytosis is shown in Figure 4B. OWE conditions in belugas displayed a larger change in phagocytosis than humans following the dive period for the 30 min (*p* = 0.075) and 5 min exposures (*p* = 0.079). OWE conditions continued to show a greater response to pressure as compared with baseline conditions in belugas for the recovery periods of the 30 min (*p* = 0.039) and single 5 min exposures (*p* = 0.041).

Inflammation samples appear to display much smaller changes in monocyte phagocytosis than either baseline or OWE samples for belugas, and look more similar to human samples for the dive periods of the 30 min and single 5 min exposures Figure 4B.

#### Effects of 1000 psi with 2 min compression/decompression (1000G) on phagocytosis

**Figure 5:**
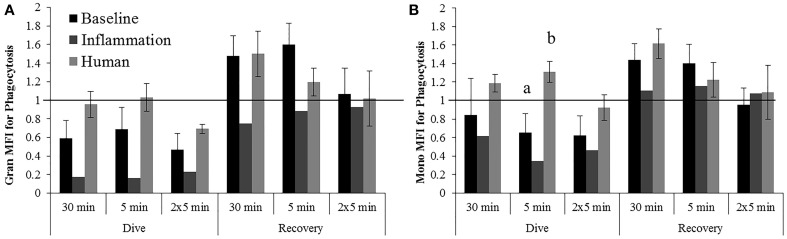
**Changes in Granulocyte (A) and Monocyte (B) phagocytosis following exposures to 1000G, measured in belugas under baseline (*n* = 4), OWE (*n* = 3) and inflammation (*n* = 2) conditions, and humans (*n* = 4)**. Data have been normalized to controls (represented by the solid line at 1) and presented as the mean change in function ± SE. Values greater than 1 indicate increased function, and values less than 1 indicate decreased function following pressure exposures. Significant differences between belugas and humans, or between conditions in belugas are indicated with letters (*p* < 0.05).

Inflammation samples display larger decreases in granulocyte phagocytosis during the dive periods following all exposures to 1000 psi as compared with baseline beluga samples or humans (Figure 5A). No apparent differences in granulocyte responses were noted during the recovery period of these exposures which differs from the increases observed in baseline samples.

The increase in monocyte phagocytosis for humans was significantly different from the decrease observed in belugas for the dive period of the 5 min exposure (Figure 5B; *p* = 0.016). Larger decreases in monocyte phagocytosis were observed in inflammation samples as compared with baseline beluga or human samples for the dive periods of all exposures to 1000 psi.

#### Effects of 2000 psi and 1000 psi with 15 s compression/decompression (2000R and 1000R) on phagocytosis

No significantly different responses to pressure were detected for exposures to 2000R. However, for exposures to 1000R (Data not shown), humans displayed significant larger changes in phagocytosis than belugas for the dive period of the 30 min exposures (Granulocytes, *p* = 0.039) and for both the dive and recovery periods of the single 5 min exposures (Monocytes, dive, *p* < 0.001; recovery, *p* = 0.016).

#### Effects of pressure exposures on CD11b expression

**Figure 6:**
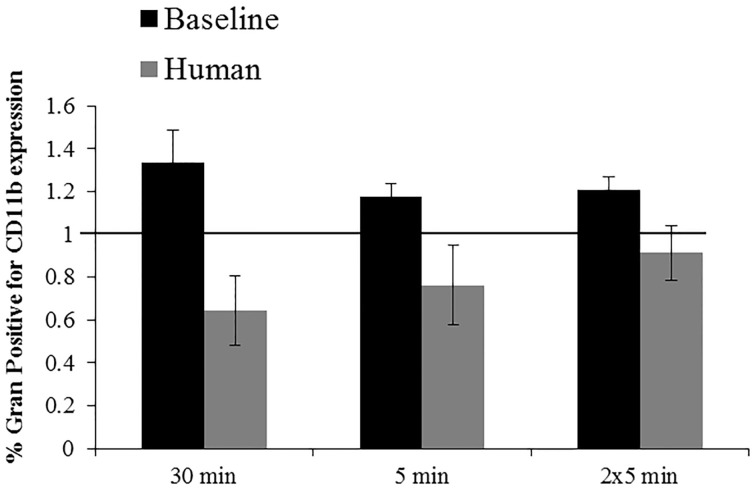
**Changes in CD11b expression following exposures to 1000R measured in beluga baseline samples (*n* = 4) and humans (*n* = 4)**. Data have been normalized to controls (represented by the solid line at 1) and presented as the mean change in function ± SE. Values greater than 1 indicate increased function, and values less than 1 indicate decreased function following pressure exposures.

No significantly different responses in the % positive granulocytes for CD11b expression were detected following exposures to 2000G, 2000R or 1000G. However, following exposures to 1000R, belugas display an increase in expression while humans display a contrasting decrease (Figure 6). Inflammation samples, display slightly smaller changes in function following the repeated 5 min exposures to 1000G as compared with other conditions in belugas (Data not shown).

## Discussion

While the dive profiles used in this study represent extreme or rare dives in the dive repertoire of belugas, results suggest that marine mammal immune cells may display altered function during diving, and characteristics of a dive play a role in determining the response of immune cells. For most dive profiles beluga cells displayed either no change or decreased phagocytic activity, with exposures to 1000 psi with rapid compression/decompression being the exception. Function of pressure exposed cells either returned to control levels or increased following the 20 min post-dive recovery periods. These changes varied however (e.g., in magnitude) between dives with different rates of compression/decompression, different target depths, and different durations. In general, larger changes were observed for dives to 2000 psi as compared with 1000 psi and for exposures with 15 s of compression/decompression as compared with 2 min and again with exposures to 1000 psi with rapid compression/decompression being the most different. In contrast, CD11b expression seemed to be less effected by simulated pressure exposures, though significant decreases in expression were detected for the repeated 5 min dives to 2000 psi with 2 min of compression/decompression, and increases were observed following exposures to 1000 psi with rapid compression/decompression. Changes in expression were larger following exposures to 2000 psi with 2 min of compression and decompression than other exposures. In addition, changes in CD11b expression were different between exposures of different durations, particularly the single and repeated 5 min exposures.

The decrease in phagocytosis observed immediately following decompression for many of the exposures was somewhat surprising, as it was expected that beluga immune cells would possess adaptations which would allow “normal” function to occur even during diving. Decreased immune function during diving, however, could be beneficial in reducing the likelihood of damage caused by aberrant immune activity or inflammatory processes. In humans, immune activity plays an important role in the development of decompression sickness (Ward et al., 1987; Nyquist et al., 2004; Barack and Katz, 2005). In particular, damage is facilitated by inflammatory processes initiated through interactions with bubbles or though endothelial damage or denaturation of host proteins (Barack and Katz, 2005). The increases in activity seen among the responses of human granulocytes may suggest an increase in reactivity of cells following a dive. In some cases, “silent bubbles” can exist in a diver without eliciting an inflammatory response and resulting in disease (Barack and Katz, 2005). Errson et al. (2002) report that studies which pre-tune the immune system by treatment with foreign proteins before a dive, lead to decreased incidence of DCS. Thus, a less reactive immune system may serve a protective function. To the best of our knowledge, tissue nitrogen saturation for diving belugas has not been reported. However, up to 300% nitrogen super saturation has been estimated for some deep diving species (Jepson et al., 2003). It is possible that “silent bubbles” exist and are more common in diving marine mammals than previously thought. Dennison et al. (2012) found gas bubbles, arising from desaturation following interruption of normal dive behavior by stranding, can be asymptomatic in live-stranded dolphins. Reduced reactivity of immune responses in marine mammals has been suggested as one mechanism by which they avoid development of decompression sickness and other dive related injuries (Ward et al., 1987; Fahlman et al., 2006). The decreases in granulocyte function observed in this study lend some support to this.

The return of values to control levels following the recovery period of the 2000G exposures suggests marine mammals cells may quickly recover from changes which occur during dives and animals may not be at greater risk of developing infection under normal conditions. Following other exposures (e.g., exposures with rapid compression and decompression) increased function was detected for the recovery periods. Increases in activity following recovery periods were also detected in belugas during stressor conditions. While we did not continue to monitor cells to see how long these changes lasted, pressure-induced activation during a dive or immediately following ascent may mean cells are more reactive to otherwise silent bubbles and could pose a health risk.

In contrast to the response of beluga cells to increased pressure exposures, humans tended to show minimal change (e.g., following exposures to 2000 psi) or increased phagocytic activity (e.g., following exposures to 1000 psi). Shiratsuchi and Basson (2004) report that extracellular pressure, associated with inflammation for example, can modulate phagocytosis in human macrophages by altering intracellular pathways. It is not unreasonable then to see much larger changes in pressure also having an effect on human monocyte phagocytosis. In some cases, however, the relationship between belugas and humans is reversed (e.g., monocytes following the 30 min exposure to 1000R). What is important about these trends is that samples drawn from belugas under baseline conditions seem to respond differently to changes in pressure than those obtained from humans. Belugas are capable of dives to depths greater than 1000 m (Heide-Jorgensen et al., 1998; Suydam et al., 2001). The deepest no-limit free dive in humans however reached only 249.6 m (herbertnitsch.com/world_records.html) with^1^ the deepest SCUBA dive reported at 318 m (scubarecords.com), though deeper dives can be achieved with special gas mixes and equipment. For example, exposure to 660 m has been achieved within a pressure chamber using Trimix (Logie and Baddeley, 1983). Thus, differences in the response of immune cells between humans and belugas may reflect different degrees of dive adaptation. Field (2000) reported that platelets from elephant seals and humans responded differently when exposed to 2800 psi pressure (2000 m) and red blood cells from deep-diving, shallow-diving and terrestrial mammals have been reported to function differently (measured as glycolytic activity) in response to pressure exposures (Castellini et al., 2001). For both platelets and erythrocytes, membrane cholesterol content has been reported to vary between marine and terrestrial mammals (Field, 2000; Williams et al., 2001) and can be important in determining cell sensitivity to changes in pressure, as cholesterol content is related to membrane fluidity. Thus, future studies should consider investigating membrane composition of immune cells.

In most cases, no significant changes in CD11b expression were observed following pressure exposures, even when changes in phagocytic activity were detected. This may suggest that pressure may have a greater mechanical effect on immune cells, and physically alter phagocytosis by altering membrane structure and fluidity, rather than affecting the activation state of cells. Additionally, there are multiple pathways by which phagocytosis can occur. CD11b, also termed CR3, is a subunit of the adhesion complex known as macrophage 1 antigen (MAC1) and facilitates phagocytosis via the alternate pathway of complement activation by binding C3bi (Murphy et al., 2008). Other pathways of phagocytosis were likely occurring in this study, and can be differentially affected by simulated pressure exposures. Thus, the phagocytosis results measured were cumulative of different pathways and it is perhaps not entirely unexpected that no apparent relationship was found between CD11b expression and phagocytosis. An important note however is that samples used for CD11b expression were also exposed to PI Staph for phagocytosis experiments, due to sample and equipment limitations and the confounding effects of PI Staph on cell activity must be considered in interpretation of results. That we did not detect further increases in CD11b expression may suggest no additive effects of pressure on top of an immune challenge such as a bacterial infection.

Changes observed following pressure exposures varied with the duration of exposure, and comparison of the response of immune cells between dive profiles revealed that altering depth and rates of compression and decompression can change the response of granulocytes and monocytes. Again, the patterns of response varied between species and with duration of exposure. For both belugas and humans, however, exposures to 1000 psi with rapid (15 s) compression/decompression appeared to be the most different from other exposures. While the rate of compression and decompression for these exposures is faster than any descent or ascent for marine mammals, the results suggest that increasing descent or ascent rates may have important implications for how immune cells respond to dive behavior. If a startle response at depth leads to a faster than normal ascent, unwanted immune activity may facilitate injury.

There are multiple examples for cetaceans in which a startle response at depth may lead to a faster than normal ascent and/or dive patterns are interupted. Behavioral changes, including changes in dive patterns can occur in response to human activities. Increased dive durations in the presence of boats have been reported for bottlenose dolphins (Constantine et al., 2004) and belugas have been reported to show extreme avoidance of ships, such as ice breakers, though a lot of variability is noted in the response of these animals to human activities (Richardson and Wursig, 1997). Sivle et al. (2012) reported changes in dive behavior of killer whales (*Orcinus orca*), long finned pilot whales (*Globicephela melas*) and sperm whales (*Physeter macrocephalus*) in response to mid and low frequency active sonar. Altered risk of bubble formation and DCS was predicted from these results (Kvadsheim et al., 2012).

The results of this work provide some evidence that changing the dive profile alters the response of immune cells. It is possible that the combination of dive characteristics that result in “normal” changes to immune function is dynamic and that belugas are susceptible to dive related disease only under specific conditions. For example, a prolonged dive to greater than normal depth can pose a greater risk for an animal undergoing wound healing or fighting an infection than an uninjured healthy individual. Because this study looked at the effects of pressure, beluga samples were only obtained from belugas resident at the Mystic Aquarium in order to avoid any confounding effect of air shipment. This population consists of two males and two females of varying age. While immune responses can be modified by many factors including age and sex, the small sample size precluded determination of differences between sexes and age groups. The pressures used represent extreme deep dives, and while it would be interesting and physiologically relevant to compare these results with exposures to much smaller pressures, this could not be done reliably with the pressure system available. Belugas are, however, capable of dives to over 1000 m, and certainly to 680 m. Thus, the information gained from this study is relevant to physiological conditions experienced by belugas.

In addition to describing the baseline response of beluga granulocytes and monocytes to pressure, we aimed to investigate the potential role of an additional physiological challenge in modifying that response utilizing samples drawn during an out of water examination (OWE) and during a period of chronic mild inflammation. Hormone data support the categorization of the OWE as a stressor. Even though the increase in norepinephrine was not statistically significant, the increase in cortisol was, as expected. Schmitt et al. (2010) reported baseline and OWE cortisol to be 2.2 ± 0.9 and 7.9 ± 1.5 μg dl^−1^ respectively, and cortisol values obtained during this study were similar. Epinephrine was detectable only for the OWE, and not during most baseline or inflammation conditions.

Phagocytosis data from OWE and inflammation conditions displayed general patterns of decreased phagocytic activity for the dive period of all exposures, with recovery periods for the 2000 psi exposures suggesting increased activity. No significant changes in CD11b expression were detected, which was similar to results from baseline conditions in belugas. However, larger changes were observed in both OWE and inflammation samples as compared with baseline samples and in some cases (particularly monocytes) these changes were more similar to those observed in humans than in baseline conditions. In humans, dive-related changes in immune function have been linked to the development of injury and disease (Brenner et al., 1999). If physiologically challenging conditions can alter the response of marine mammal cells such that it resembles the response of human cells, it is possible that under such conditions marine mammals become more susceptible to dive related pathologies. However, consequences of decreased immune function are not always negative, and conversely increased immune function is not always protective. What is of concern, is that responses occurring in belugas during stressor conditions are different from responses which occur under baseline conditions, suggesting that there are important trade-offs between dive behavior and immune function, and that the relationship between behavior and health is dynamic.

While the belugas in this study were either born in or housed in aquaria for the majority of their lives, it is possible that the response of wild belugas will be different due to previous diving experience. Dive experience and training have been shown to aid in development of dive capabilities (Ferretti and Costa, 2003; Lander et al., 2003; MacArthur et al., 2003) and result in acclimatization which reduces incidence and severity of decompression sickness (Su et al., 2004). Lymphocytes from wild belugas respond differently to pressure exposures (Thompson and Romano, unpublished data) and it is not unreasonable that other immune cells would follow suit.

Additionally, this study used an entirely *in vitro* approach, which allowed us to focus on the effects of changes in pressure *per se* on immune function. However, there are many physiological changes which occur during diving that can also affect immune function, such as changes in temperature, oxygen availability or levels of circulating endocrine hormones that control the dive response. The temperature of the pressure chamber was monitored and held to 37°C ± 2°C in order to avoid confounding effects of temperature changes on cell function, though the available equipment did not allow for the measurement of oxygen throughout the dive. Changes in oxygen availability within the chamber may have contributed to the measured results. Additionally, immune responses involve complex pathways and complicated interactions between cells and blood components, including redundancies which may compensate for pressure induced changes observed here. In some cases, granulocytes and monocytes displayed different responses to pressure even though the same function was being measured. While conclusions on the significance of this finding are beyond the scope of this work, redundancies with the immune system (e.g., different cell types performing the same function) may play an important role in maintaining immune responses during diving. Future studies possibly combining dive behavior data from tagging with blood sampling would provide a more accurate picture of what occurs during natural diving. In addition, other measures of immune function should be considered, including radical oxide production which is important for destroying bacteria, but also in endothelial damage and facilitating inflammatory responses, or complement activation, which plays an important role in the development of DCS.

More work is needed to better describe the complicated relationship between diving, stress and immune function in marine mammals and actual implications of immune function changes during diving in marine mammals remain speculatory due to limited prior information and contextual studies. However, such information is important in understanding potential sub-lethal impacts of anthropogenic activities on marine mammal health, and long term health trends for marine mammal species. This work describes for the first time, the response of immune cells to increased pressure for any marine mammal species and provides evidence suggesting that one mechanism through which anthropogenic activity could impact marine mammal health is by altering the response of immune cells during diving, either by acting as a stressor or altering behavior. Results demonstrate that beluga granulocytes and monocytes may respond differently to changes in pressure than human cells, and that this response can be modified by changing the depth, duration and rates of compression and decompression for the dive, or by physiological status.

### Conflict of interest statement

The authors declare that the research was conducted in the absence of any commercial or financial relationships that could be construed as a potential conflict of interest.

## Footnotes

^1^www.Herbertnitsch.com. World Records. [Accessed August 2013].

## Abbreviations

DCS: decompression sickness
PI Staph: propidium iodide labeled Staphylococcus aureus
G: gradual compression and decompression occurring over 2 min
R: rapid compression and decompression occurring over 15 s
MFI: mean fluorescence intensity
PBS: phosphate buffered saline
HBSS: Hanks balanced salt solution
PMA: phorbol myristate acetate
FITC: fluorescein isothiocyanate.
